# The number of women who would need to be screened regularly by mammography to prevent one death from breast cancer

**DOI:** 10.1258/jms.2011.011134

**Published:** 2011-12

**Authors:** Valerie Beral, Maggie Alexander, Stephen Duffy, Ian O Ellis, Rosalind Given-Wilson, Lars Holmberg, Sue M Moss, Amanda Ramirez, Malcolm W R Reed, Caroline Rubin, Patsy Whelehan, Robin Wilson, Kenneth C Young

**Affiliations:** Cancer Epidemiology Unit, University of Oxford, Richard Doll Building, Roosevelt Drive, Oxford OX3 7LF; Breakthough Breast Cancer, London; Centre for Cancer Prevention, Queen Mary University of London, Wolfson Institute of Preventive Medicine, Charterhouse Square, London EC1M 6BQ; Dept Histopathology, City Hospital Campus, Nottingham University Hospitals, Hucknall Rd, Nottingham NG5 1PB; St. George's Healthcare NHS Trust, London; King's College, London; Centre for Cancer Prevention, Queen Mary University of London, Wolfson Institute of Preventive Medicine, Charterhouse Square, London EC1M 6BQ; King's College London Promoting Early Presentation Group, National Clinical Lead for Cancer Patient Information, Institute of Psychiatry, King's College, London; University of Sheffield, The Medical School, Sheffield, S10 2RX; University Hospital Southhampton NHS Foundation Trust, Southampton, Hants SO16 6YD; University of Dundee, Ninewells Hospital and Medical School, Dundee DD1 9SY; The Royal Marsden NHS Foundation Trust, London; National Co-ordinating Centre for the Physics of Mammography, Royal Surrey County Hospital, Guildford GU2 7XX

## Abstract

The number of women who would need to be screened regularly by mammography to prevent one death from breast cancer depends strongly on several factors, including the age at which regular screening starts, the period over which it continues, and the duration of follow-up after screening. Furthermore, more women would need to be INVITED for screening than would need to be SCREENED to prevent one death, since not all women invited attend for screening or are screened regularly. Failure to consider these important factors accounts for many of the major discrepancies between different published estimates. The randomised evidence indicates that, in high income countries, around one breast cancer death would be prevented in the long term for every 400 women aged 50–70 years regularly screened over a ten-year period.

Randomized evidence has shown that regular mammographic screening of women aged between about 50 and 70 years reduces mortality from breast cancer.^1–4^ The absolute reduction in mortality achieved by regular screening, and hence the number of women who would need to be screened regularly over a period of time to prevent one death from breast cancer, depends on several factors. These include:

- women's ages when screened, as incidence and mortality rates for breast cancer increase with age;
- the period over which regular screening occurs;
- the duration of follow-up after a period of screening, as deaths from the disease still occur two to three decades after an initial diagnosis of breast cancer,^4,5^ and treatment-associated reductions in mortality from breast cancer continue into at least the second decade after the treatment;^6,7^
- whether estimates relate to numbers of women who would need to be invited for screening (which is what randomized trials mostly address) or whether estimates relate to the number who would need to accept an invitation and be screened regularly (as not all women who are invited for screening accept the invitation, or are screened regularly);
- how often breast cancer would have been diagnosed in the absence of screening. This is changing over time, with changes in the underlying incidence of the disease and the increasing awareness of breast cancer, together with the increasing need to investigate suspect breast abnormalities.

Estimates in the table are all derived from randomized trial results. Section A gives the estimated numbers who would need to be INVITED for screening to prevent one death from breast cancer.^2,3^ The US Preventive Services Task Force^2^ subdivided their estimates by women's ages at first invitation. As expected, the estimated numbers that would need to be invited for screening to prevent one death from breast cancer is substantially greater for younger than for older women – for example, they estimate that approximately five times as many women aged 40–49 than 60–69 years would need to be invited for screening to prevent one death from breast cancer (1904 vs 377, see Table 1). These estimates are based on breast cancer deaths occurring during the first 13 or so years after women were first invited for screening.

**Table 1:** Estimated numbers of women that would need to be (A) invited for screening and (B) screened to prevent one death from breast cancer

Published reference	Age at: (A) first invitation, (B) first screen	Period of (A) invitation or (B) screening*	Follow-up after: (A) first invitation, (B) first screen	Estimated absolute numbers (A) to invite or (B) to screen, to prevent one death from breast cancer (& 95% CI)
A. Numbers needed to INVITE for screening to prevent one breast cancer death				
US Preventive Services Task Force^2^	40–49 years	∼7 years	∼13 years	1904 (929–6378)
	50–59 years	∼7 years	∼13 years	1339 (322–7455)
	60–69 years	∼7 years	∼13 years	377 (230–1050)
Gotzsche & Nielsen^3^	not stated^†^	unclear^†^	unclear^†^	2000 (CI not stated)
B. Numbers needed to SCREEN to prevent one breast cancer death				
Tabar et al^4‡^	40–74 years	7 years	10 years	1303 (621–13169)
			20 years	577 (370–1315)
			29 years	519 (336–1144)
ACBCS^5^	50–70 years	10 years	>30 years	400 (CI not stated)

*The average time between each INVITATION for screening in the 8 trials reviewed by the US Preventive Services Task Force^2^ ranged from 12 months to 2½ years. The average time between each SCREEN in the Swedish Two County trial^4^ was 33 months, and the time between each screen assumed to be 36 months by the Advisory Committee on Breast Cancer Screening (ACBCS)^5^

^†^It is unclear whether Gotzsche and Nielsen's estimate is for invitations over a 10-year period or for follow-up over 10 years or both (see text); and the age group to which their estimate applies is not stated, but may have been heavily weighted by women aged under 50 years^3^

^‡^Based on breast cancer deaths assessed by the Swedish Overview Consensus Committee

Gotzsche and Nielsen^3^ state ‘for every 2000 women invited for screening throughout 10 years, one will have her life prolonged’. However, the authors do not state the age group to which their estimate applies, and it is unclear whether ‘throughout 10 years’ refers to the period of invitation, the period of follow-up, or both.

Section B of the table gives the estimated numbers needed to SCREEN to prevent one death from breast cancer.^4,5^ Recent data published by Tabar *et al*.^4^ from two randomized trials in Sweden (the ‘Two County’ trial) illustrate the importance of duration of follow-up, as fewer than half the deaths from breast cancers diagnosed during the screening period occurred in the first 10 years of follow-up after first randomization. They estimate that, after 29 years of follow-up for women aged 40–74 years at first screen, 519 would need to be screened over a seven-year period and that approximately 400 such women would need to be screened over a 10-year period, to prevent one death from breast cancer.^4^

Only 85% of those invited for screening in the Swedish Two County trial accepted any single invitation and fewer still were screened regularly.^4^ Furthermore, some women in the control arm of the trial were screened (outside the trial) during the study period. Because randomized trials generally assess the reduction in mortality associated with being invited for screening, it follows that results from such trials do not provide a direct measure of the reduction in mortality associated with being screened. Trial results for women invited for screening are inevitably diluted by the misclassification of women's true screening history, both in the intervention arm and in the control arm. The International Agency for Research on Cancer^1^ used randomized trial results to estimate the relative risk for breast cancer mortality associated both with being invited for screening and with being screened, adjusting for the measurement error associated with misclassifying women in the trials. As would be expected, they found a greater relative reduction in breast cancer mortality associated with being screened than with being invited for screening.^1^ (As would also be expected, the International Agency for Research on Cancer^1^ and the US Preventive Services Task Force^2^ found similar relative risks for breast cancer mortality among women aged 50–69 years who were INVITED for screening, as the same trials were included in the analyses for both reports.)

The Advisory Committee on Breast Cancer Screening (ACBCS)^5^ estimated that 400 women in England aged 50–70 years would need to be screened regularly over a 10-year period to prevent one death from breast cancer during the 30 or so years after women's first screen (i.e. including the 20 or so years after women's last screen). The ACBCS's estimate was calculated by applying relative risks for screened women, derived by the International Agency for Research on Cancer,^1^ to breast cancer deaths in England in 2003. Although the methods used by the ACBCS^5^ were different to those used by Tabar *et al*.,^4^ the estimate that 400 women aged 50–70 in England would need to be screened regularly over 10 years to prevent one death from breast cancer is the same as that made by Tabar *et al*. that 400 women aged 40–74 years in Sweden would need to be screened over a 10-year period to prevent one death from breast cancer. Furthermore, only about three-quarters of the women in England who are invited for screening accept the invitation,^5^ and so the US Preventive Services Task Force's estimates of the numbers aged 50–69 years who would need to be invited for screening are broadly consistent with the ACBCS estimates for the number of women who would need to be screened, after allowing for the incomplete uptake of screening by those invited and differences in the duration of follow-up.

When inviting women for screening, as the NHS Breast Screening Programme does, it is appropriate to provide statistics on numbers needed to SCREEN to prevent one breast cancer death. (If the number needed to invite for screening were quoted, it might be taken to imply that the invitation itself was sufficient to prevent death from breast cancer.) Also, estimates of the number needed to screen to prevent one death from breast cancer needs to reflect the long-term effect of screening, not just what happens in the first decade or so after an initial screen (as screening in a given 10-year period prevents more deaths after that 10-year period ends than during it).

In summary, based on randomized evidence, it is reasonable to conclude that, in high income countries, around one breast cancer death would be prevented in the long term for every 400 women aged 50–70 years regularly screened over a ten-year period. The numbers needed to screen to prevent one death from breast cancer depend strongly on the age at which regular screening starts, the period over which it continues, and the duration of follow-up after screening. Failure to consider these important influences on breast cancer mortality accounts for many of the major discrepancies between different published estimates.

## Declarations

All authors are independent members of the Department of Health's Advisory Committee on Breast Cancer Screening in England (Chair: VB).

## References

[1] International Agency for Research on Cancer Breast cancer screening. IARC Handbooks of Cancer Prevention. Volume 7. IARsC press, Lyon, 2002

[2] Nelson HD, Tyne K, Naik A, Screening for breast cancer: systematic evidence review update for the U.S. Preventive Services Task Force. Ann Intern Med 2009;151:727–W242 19920273 10.1059/0003-4819-151-10-200911170-00009 PMC2972726

[3] Gøtzsche PC, Nielsen M Screening for breast cancer with mammography. Cochrane Database of Systematic Reviews 2009 10.1002/14651858.CD001877.pub3 19821284

[4] Tabár L, Vitak B, Chen TH, Swedish two-county trial: impact of mammographic screening on breast cancer mortality during 3 decades. Radiology 2011;260:658–63 21712474 10.1148/radiol.11110469

[5] Advisory Committee on Breast Cancer Screening Screening for breast cancer in England: past and future. *J Med Screen* 2006;**13**:59–61 and also http://www.cancerscreening.nhs.uk/breastscreen/publications/nhsbsp61.html 10.1258/096914106777589678 16792825

[6] Early Breast Cancer Trialists' Collaborative Group (EBCTCG) Relevance of breast cancer hormone receptors and other factors to the efficacy of adjuvant tamoxifen: patient-level meta-analysis of randomised trials. Lancet 2011;378:771–84 21802721 10.1016/S0140-6736(11)60993-8 PMC3163848

[7] Early Breast Cancer Trialists' Collaborative Group (EBCTCG) Effect of radiotherapy after breast-conserving surgery on 10-year and 15-year breast cancer death: meta-analysis of individual patient data for 10,801 women in randomised trials. Lancet 2011;378:1707–16 22019144 10.1016/S0140-6736(11)61629-2 PMC3254252

